# The Evolution of Fretting Wear Behavior and Damage Mechanism in Alloy 690TT with Cycle Number

**DOI:** 10.3390/ma13102417

**Published:** 2020-05-25

**Authors:** Long Xin, Yongming Han, Ligong Ling, Weidong Zhang, Yonghao Lu, Tetsuo Shoji

**Affiliations:** 1 National Center for Materials Service Safety, University of Science and Technology Beijing, Beijing 100083, China; ymhan@ustb.edu.cn (Y.H.); lingligong2008@163.com (L.L.); zwd@ustb.edu.cn (W.Z.); tshoji@fri.niche.tohoku.ac.jp (T.S.); 2 Nuclear and Radiation Safety Center, Beijing 102401, China; 3 Frontier Research Initiative, New Industry Creation Hatchery Center, Tohoku University, 6-6-10, Aramaki Aoba, Aoba-ku, Sendai 980-8579, Japan

**Keywords:** fretting running status, fretting induced fatigue, fretting induced wear, Alloy 690TT, glaze layer, TTS, cycle number

## Abstract

The evolution of fretting wear behavior and damage mechanism in Alloy 690TT with cycle number was investigated via laser scanning confocal microscopy (LSCM), scanning electron microscopy (SEM), focus ion beam (FIB), and transmission electron microscopy (TEM). The results showed that the fretting running status underwent a transition from partial slip and mixed stick-slip to final gross slip with the transformation of Ft–D curves from the ellipse to the parallelogram. The coefficient of friction (COF) experienced three drops throughout the fretting process, which indicated the transformation from high-friction wear to low-friction wear. The first drop was due to the transition from two-body to three-body contact. The second and third drops were mainly related to the evolution of the glaze layer from a localized distribution to completely covering the whole contact surface. The competition between fretting induced fatigue cracking (FIF) and fretting induced wear (FIW) ran through the entire fretting wear process. Before the 1.2 × 10^4^th cycle, the fatigue crack growth was faster than wear, and FIF won the competition. As the fretting cycle continued to increase, the wear velocity was obviously faster than that of FIF, which indicated that FIW defeated FIF. The tribologically transformed structure (TTS) participated in the competition between FIF and FIW. The gain boundaries and dislocations in the TTS were a suitable pathway for crack initiation and propagation and oxygen permeation.

## 1. Introduction

Fretting wear is a material damage in contact surfaces, which is induced by small movements between two contact bodies [1,2]. As the contact loading conditions (such as normal force, tangential displacement amplitude) change, the fretting running characteristics vary between partial slip and gross sliding [3]. When a situation is viewed over time, the shape of the fretting loop and the evolution of the tangential force-relative slip relationship dominate the fretting maps [4]. Furthermore, four fretting regimes (stick, partial slip, mixed, and gross slip) appear in the fretting map [5,6,7]. If the normal force and/or fretting stoke is small, partial slip occurs. In a partial slip regime (PSR), the sticking in the center and slipping on the edge exist at the contact surface and cracks initiate at the interface [8]. If contacting surfaces are subjected to a lower normal force and longer strokes, gross slip will take place. In a gross sliding regime (GSR), severe wear damage and oxidation occur and the crack initiation is limited [1,9]. With the increase in normal load and/or the decrease in stroke, a mixed fretting condition will be formed. The mixed fretting regime (MFR) is the most complex, and involves surface adhesion, abrasion, delamination, and crack nucleation and propagation, which results in a combination of sticking and slipping throughout the wear track [10].

The heat exchanger tubes in the steam generator (SG) of nuclear power plants (NPPs) are widely produced by alloy 690TT. Fretting wear occurs between the tube and anti-vibration components due to the flow-induced vibration [11]. In view of the demand for a long-time service of 40–60 years in NPPs, the estimation of tube residual lifetime research is performed on the evaluation of the wear coefficient in the work-rate model [12,13,14]. However, the surface and subsurface stress and strain distribution can be significantly changed by varied geometry over time, resulting in the formation of damaged structures with a varying cycle number [15,16,17]. The third body layer (TBL) can be formed on the wear surface of Fe-Cr-Ni alloys, which protects the matrix from being further oxidized [18]. In the fretting process, the TBL can be broken, due to the relative sliding between frictional pairs with an increase in the cycle number [19], which improves the oxidation rate of the materials. However, few studies have been carried out to directly evaluate the evolution of the microstructure and wear mode of alloy 690 with cycle number.

Therefore, it is necessary to accumulate more experimental test data on the fretting wear performance and the failure structure with the number of cycles to reveal the wear mechanisms, which is the objective of this study.

## 2. Materials and Methods

The samples used in the fretting wear tests were a Type 304 stainless steel (SS) ball (Jiuli Group Co., Ltd., Huzhou, China) and alloy 690TT plate (Jiuli Group Co., Ltd., Huzhou, China). The 304 stainless steel is an anti-vibration component [20] which is used in CANDU nuclear power plants. The diameter, hardness, and the surface roughness (Ra) of the 304SS ball was 10 mm, 200 HV, and 0.4 μm, respectively. The alloy 690TT plate sample was derived from the corresponding tube and mechanically polished. After that, the plate sample had a hardness of ~235 HV and a surface roughness of 0.04 μm. The chemical composition and mechanical properties of the test materials are shown in Table 1 and Table 2, respectively. 

**Figure 1:**
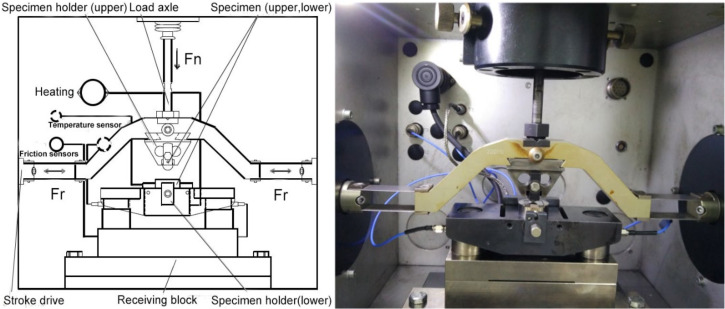
Schematic diagram of the fretting wear test rig.

The fretting wear tests were carried out by using a commercial friction and wear tester (SRV-IV) with a ball-on-plate configuration. The detailed assembly of this tester can be found in our previous studies, as shown in Figure 1 [21,22]. The test parameters can be recorded online in real time. The coefficient of friction (COF) and friction force (Ft)-displacement (D) curves can be obtained. Tests were performed three times under each condition to ensure result repeatability. The experimental parameters were selected as a normal load of 100 N, a displacement amplitude of 75 μm, and a frequency of 20 Hz with a cycle number range from 3000 to 36,000. The test environment was in air at 320 °C with a relative humidity of ~30%. Fretting tests were performed at a normal load of 100 N, which is slightly higher than the actual range of a normal load (10–90 N [23,24]) in a steam generator, due to fretting behavior, in order to produce the accelerated results. The temperature of 320 °C is located within the actual range of temperatures (270–330 °C [25]) in a steam generator.

The wear volume and the profiles of wear scars were determined by using laser scanning confocal microscopy (LSCM, Olympus LEXT OLS4000, Olympus, Tokyo, Japan). Prior to each test and wear volume measurement, the specimens of worn alloy 690TT were acoustically cleaned in acetone and then alcohol for 10 min and dried with compressed air. Worn surfaces and subsurface morphologies were examined using scanning electron microscopy (SEM, Zeiss Auriga, SE2 and InLence model, Carl Zeiss, Oberkochen, Germany) with an energy dispersive spectroscopy (EDS) detector. The transmission electron microscopy (TEM, Tecnai G^2^ F20, FEI, Portland, OR, USA) thin foils were prepared by the focus ion beam (FIB, Helios Nanolab 600i, FEI, Portland, OR, USA) technique. Detailed microstructure characterization was performed on TEM equipment (Tecnai G^2^ F20, FEI, Portland, OR, USA) operating at 200 kV. The imaging of bright field (BF), dark field (DF), and EDS were taken in scanning transmission electron microscopy (STEM, model, conpany, city, country) mode, and selected area electron diffraction (SAED, model, conpany, city, country) patterns were taken with TEM equipment. 

## 3. Results

### 3.1. Friction and Wear Data

**Figure 2:**
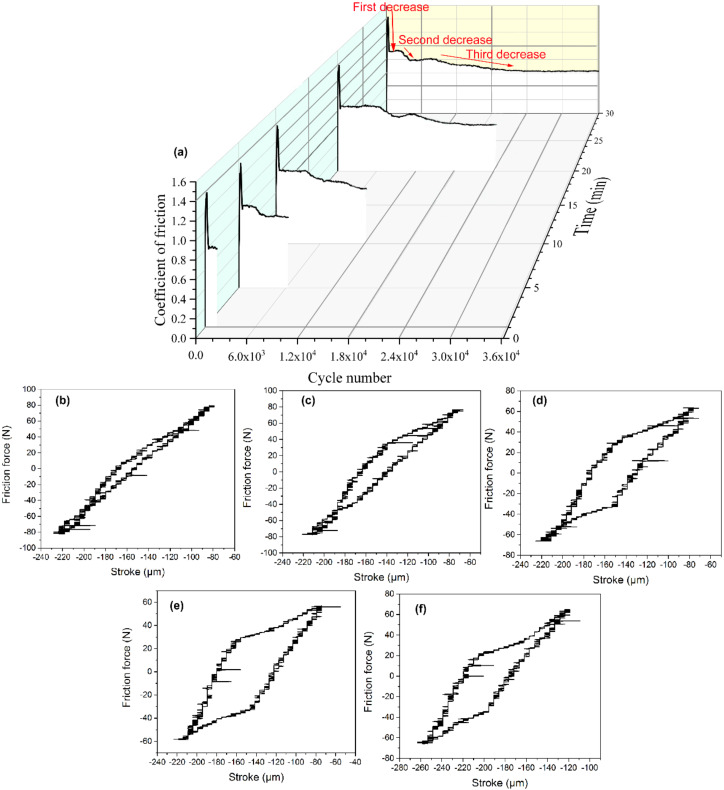
(**a**) The change in the COF as a function of cycle number with the typical Ft–D curves at a cycle number of (**b**) 1.2 × 10^3^, (**c**) 6 × 10^3^, (**d**) 1.2 × 10^4^, (**e**) 2.4 × 10^4^, and (**f**) 3.6 × 10^4^.

Figure 2 shows the change in the coefficient of friction (COF) with the number of cycles and the friction force to displacement (Ft–D) curves when the COF is stable. As shown in Figure 2a, the COF rapidly rises to a rather high value, about 1.4, during the running-in stage in the first few hundred cycles. After the running-in period, the COF drops to a stable value of 0.92 for the first time. When the fretting reaches 3000 cycles, the COF is always maintained at this stable value. When the fretting runs between the 3000th and 6000th cycle, the COF remains stable for a period and then drops to a stable value of 0.80 for the second time. After about the 8000th cycle, the COF decreases to another stable value, around 0.62, for the third time, indicating the transformation from high-friction wear to low-friction wear. The third decrease of the COF takes a longer time. As shown the Ft–D diagrams in Figure 2b–f, the transition from ellipse to parallelogram is observed with the increase in the cycle number.

**Figure 3:**
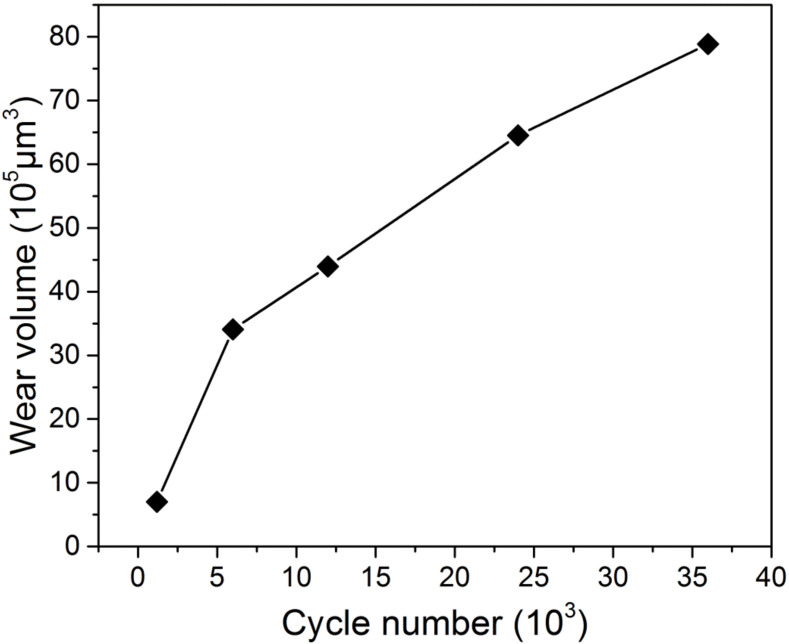
The wear volume of alloy 690TT as a function of cycle number.

**Figure 4:**
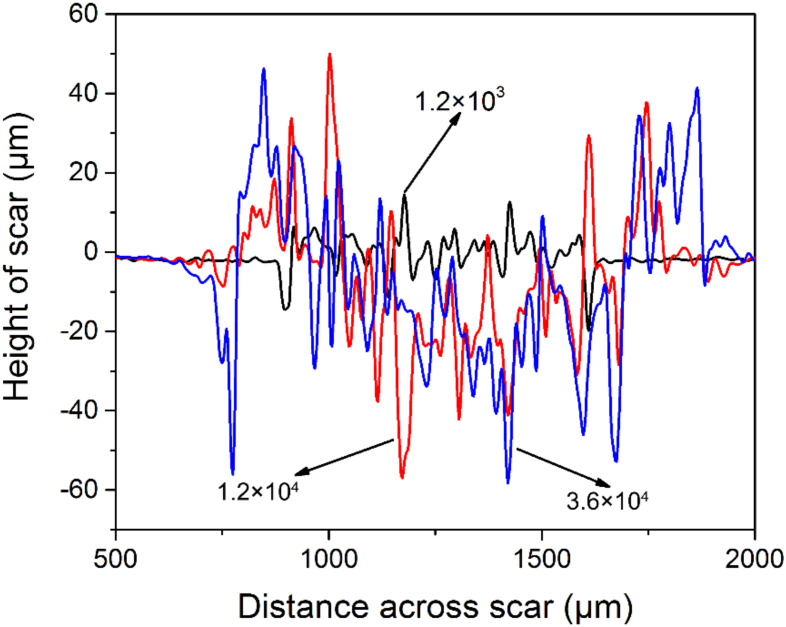
The typical cross-sectional profile of worn scars of alloy 690TT after the cycle numbers of 1.2 × 10^3^, 1.2 × 10^4^, and 3.6 × 10^4^.

Figure 3 shows the wear volume of alloy 690TT as a function of cycle number. It can be found that the wear volume gradually increases from 7.03 × 10^5^ μm^3^ to 78.859 × 10^5^ μm^3^ when the cycle number changes from 1.2 × 10^3^ to 3.6 × 10^4^. The growth rate of the wear volume also gradually slows down. Figure 4 shows the typical cross-sectional profile of worn scars after the cycle numbers of 1.2 × 10^3^, 1.2 × 10^4^, and 3.6 × 10^4^. It can be seen that wear depth and width gradually increase with the increase in cycle number. The maximum wear depth increases from 20 μm to 59 μm as the cycle number increases from 1.2 × 10^3^ to 3.6 × 10^4^.

### 3.2. Wear Surface

**Figure 5:**
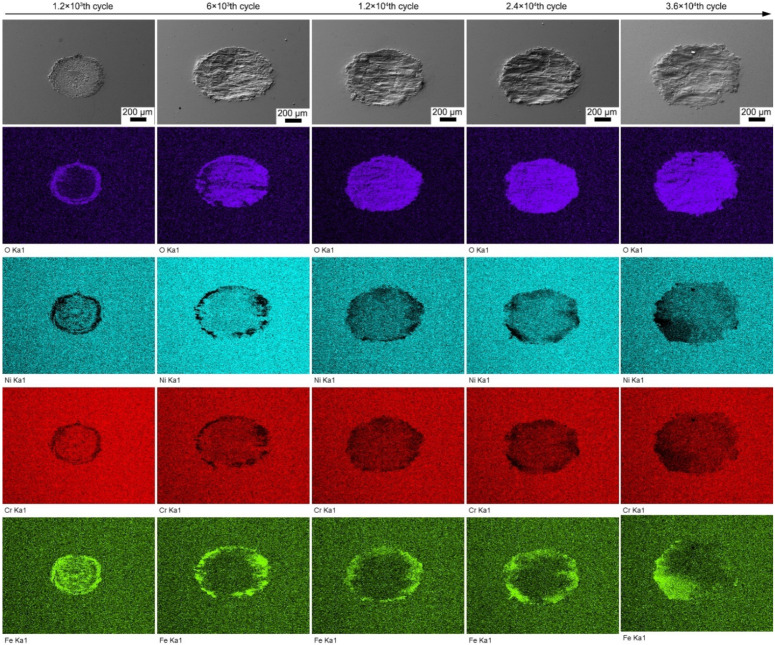
The overall SEM images of wear scars of alloy 690TT with the elemental mapping of O, Ni, Cr, and Fe at different cycle numbers.

Figure 5 shows the overall SEM images of the wear scars of alloy 690TT with the elemental mapping of O, Ni, Cr, and Fe at different cycle numbers. At the 1.2 × 10^3^th cycle, the wear morphology displays partial slip characteristics with a sticking zone in the center and an annular microslip zone at the edge. O is enriched in the microslip zone, while Fe is enriched in both areas. As the cycle number increases from 1.2 × 10^3^ to 6 × 10^3^, the sticking zone disappears. O is almost completely concentrated on the worn surface, while Fe is enriched at the edge of the worn surface. As the cycle number increases further, O is completely enriched on the worn surface, while iron is still enriched at the edge of the wear scar.

**Figure 6:**
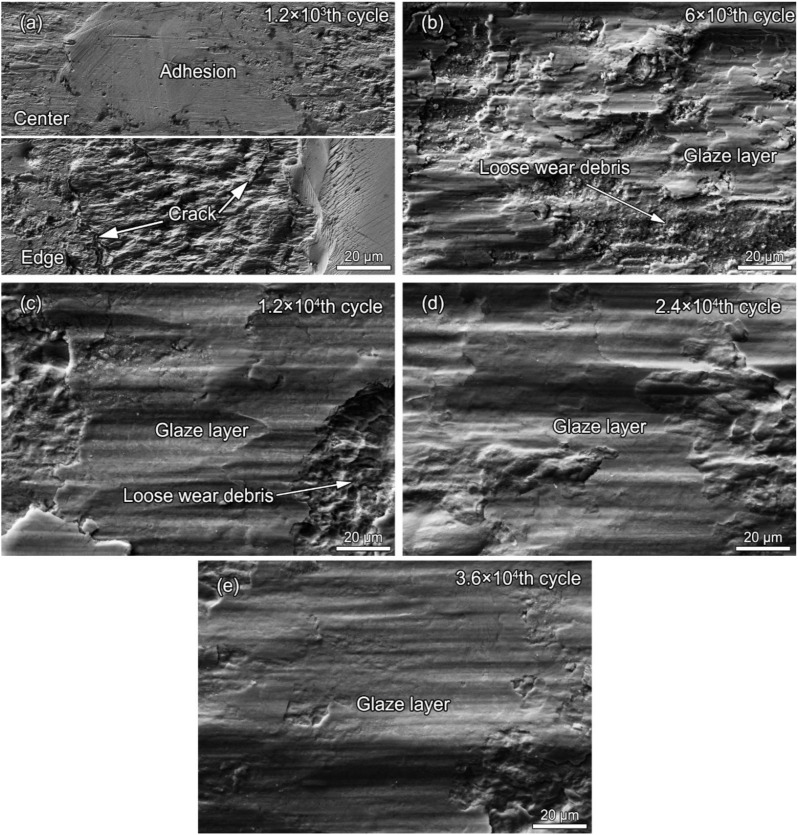
The detailed SEM surface morphologies of worn scars of alloy 690TT after fretting cycles of (**a**) 1.2 × 10^3^, (**b**) 6 × 10^3^, (**c**) 1.2 × 10^4^, (**d**) 2.4 × 10^4^, and (**e**) 3.6 × 10^4^.

Figure 6 shows the detailed SEM surface morphologies of worn scars after different fretting cycles. As shown in Figure 6a, after the 1.2 × 10^3^th fretting cycle, a hardly damaged adhesion zone still exists in the central area of the contact surface. At the same time, cracks are observed at the edge of the contact surface. As shown in Figure 6b, after the 6 × 10^3^th fretting cycle, the central adhesion zone disappears, while some local glaze layers and loose wear debris are present on the worn surface. As shown in Figure 6c, as the fretting cycle increases to 1.2 × 10^4^, a larger area of the glaze layer is gradually formed, and loose wear debris gradually disappears. As the number of fretting cycles continues to increase, the wear surface is gradually covered by the glaze layer, and loose abrasive debris is hardly seen, as shown in Figure 6d,e.

### 3.3. Wear Subsurface

**Figure 7:**
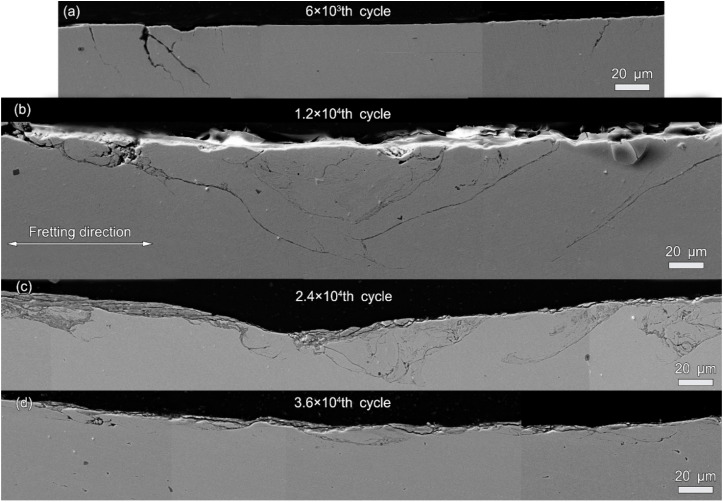
The SEM images of cross-sections of alloy 690TT after fretting cycles of (**a**) 1.2 × 10^3^, (**b**) 6 × 10^3^, (**c**) 1.2 × 10^4^, and (**d**) 2.4 × 10^4^.

Figure 7 shows the SEM images of cross-sections of alloy 690TT after different fretting cycles. As shown in Figure 7a, after the 1200th fretting cycle, fatigue cracks are formed at the edge of the wear surface. The cracks propagate in the direction of depth, and the angle between the crack and the wear surface is 50–85°. As shown in Figure 7b, after the 6 × 10^3^th fretting cycle, the number of cracks increases sharply, and the length of the cracks increases significantly, up to 180 μm. The number of cracks in the contact center increases, and the angle between the crack and the surface decreases. As shown in Figure 7c, as the fretting cycle increases to 1.2 × 10^4^, a larger grinding pit is formed and the number and length of cracks are reduced. The angle between the crack propagation direction and the wear surface is further reduced. Some cracks are connected to each other, which may cause the material to fall off. As the number of fretting cycles continues to increase, the number of cracks continues to decrease. The crack propagation direction is parallel to the fretting direction, as shown in Figure 7d.

**Figure 8:**
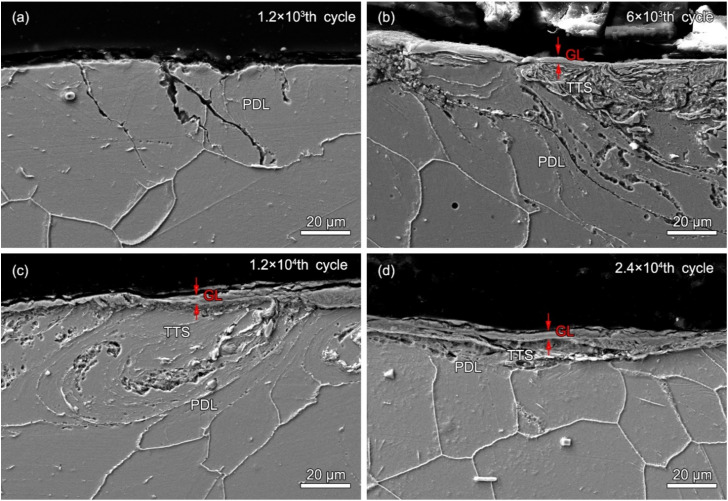
The local cross-sectional SEM images of the wear scars of alloy 690TT after fretting cycles of (**a**) 1.2 × 10^3^, (**b**) 6 × 10^3^, (**c**) 1.2 × 10^4^, and (**d**) 2.4 × 10^4^.

Figure 8 shows the local cross-sectional SEM images of the wear scars of alloy 690 after different fretting cycles. As shown in Figure 8a, after the 1.2 × 10^3^th fretting cycle, transgranular cracks are observed in the cross-section. A plastic deformation layer (PDL) is formed, which is indicated by the deformed grain boundary. As shown in Figure 8b, after the 6000th fretting cycle, a glaze layer (GL) is formed with a thickness of 2–5 μm. There is a tribologically transformed structure (TTS) just under the GL. In the TTS layer, the grain boundaries cannot be seen with SEM imaging. The PDL can be clearly observed just under the TTS layer. The grain boundaries in the PDL are stretched along the fretting direction. The cracks run within the TTS and PDL. As shown in Figure 8c, as the fretting cycle increases to 1.2 × 10^4^, the thickness of the GL is increased to 4–10 μm. There are cracks within the TTS and PDL. As shown in Figure 8d, as the number of fretting cycles continues to increase, the thickness of the GL slowly increases to 5–14 μm. However, the area of the TTS and PDL are reduced. 

**Figure 9:**
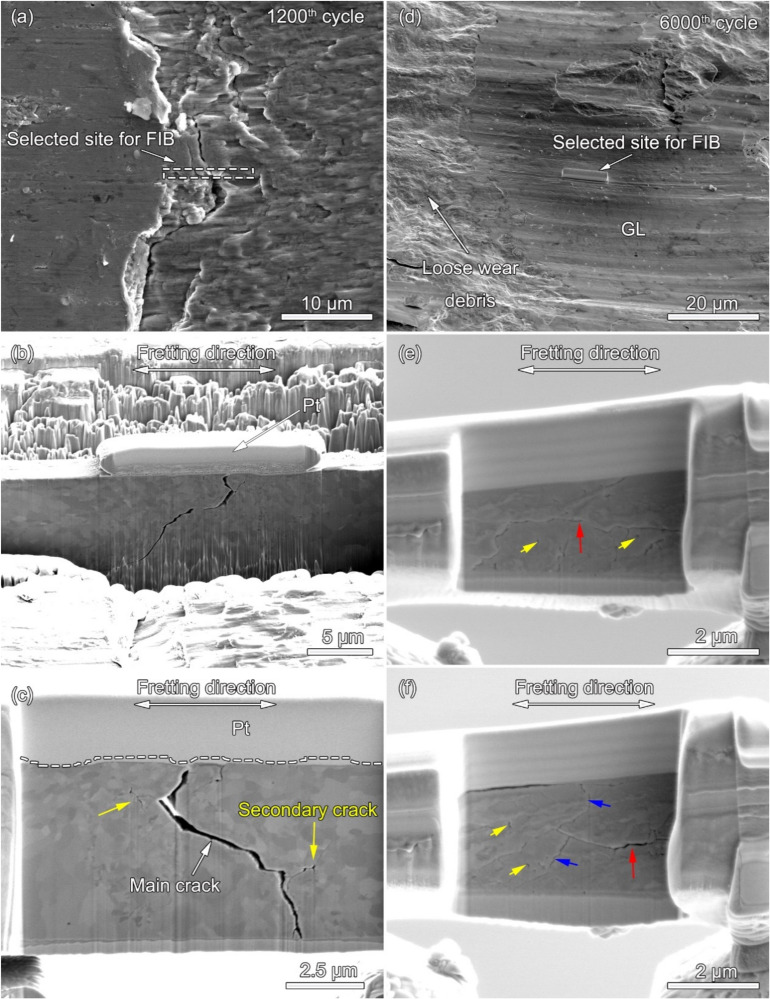
The SEM images during the FIB process: (**a**) The magnified image of the wear scar at the 1200th cycle. (**b**) The isolated membrane during the FIB process with a deposited Pt layer to protect the surface feature. (**c**) The cross-section after the final polishing. (**d**) The magnified image of the wear scar at the 6000th cycle. The cross-section after (**e**) 45 min and (**f**) 90 min of polishing.

Figure 9a shows the magnified image of the wear scar at the 1200th cycle. The selected site for the FIB is located at the crack just at the interface between the sticking region and the micro-slip region, which is indicated by the dashed rectangle. Figure 9b shows the isolated membrane during the FIB process with a deposited Pt layer to protect the surface feature. It can be seen that the grain refinement occurs in the cross-section with some cracks. Figure 9c is the SEM image showing the cross-section after the final polishing during the FIB process. It can be found that the main crack firstly propagates into a depth with an angle of 85° between the crack and the wear surface. Then the crack propagation path turns 180° and gradually into the deep subsurface. Additionally, there are some secondary cracks linked with the main crack, which is indicated by the yellow arrows. Figure 9d shows the magnified image of the wear scar at the 6 × 10^3^th cycle. It can be found that the GL and the loose wear debris layer coexist on the worn surface. The GL is chosen as the selected site for the FIB, which is protected by the Pt layer. Figure 9e shows the cross-section after 45 min of polishing during the FIB process. It can be seen that there are many cracks and voids in the subsurface, indicated by the red and yellow arrows. The delamination cracks are parallel to the wear surface, which is indicated by the red arrow. Additionally, the fatigue cracks are at an angle to the wear surface and some of them have no source. Figure 9f shows the cross-section after 90 min of polishing during the FIB process. It can be found that the delamination cracks (red arrows), fatigue cracking (blue arrows), and voids (yellow arrows) appear simultaneously.

**Figure 10:**
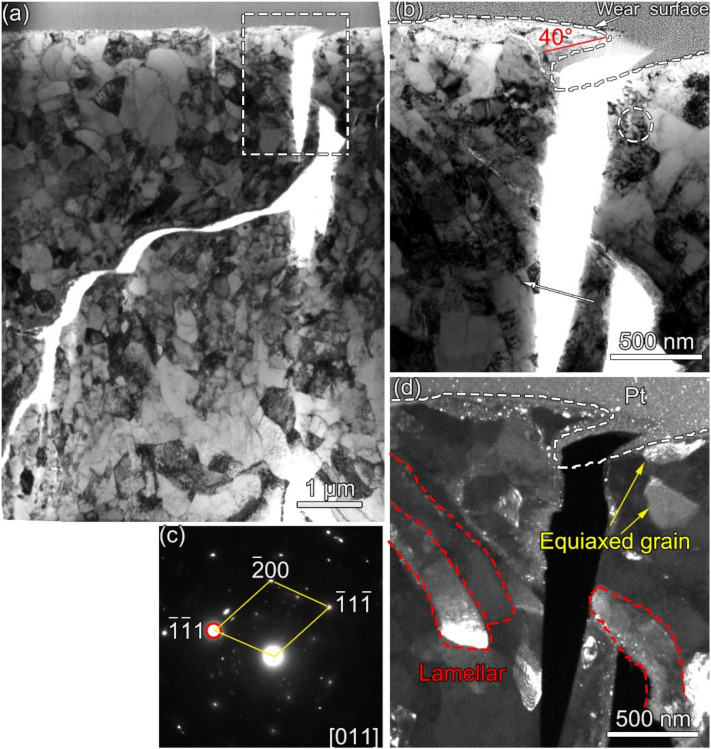
(**a**) An overview cross-sectional BFTEM image of the wear scar at the 1200th cycle after the final polishing of the FIB process. (**b**) The magnified image from the rectangle in (**a**). (**c**) The indexed SAED taken from the dashed circle in (**b**). (**d**) The corresponding DFTEM image taken from the red site in (**c**).

Figure 10a shows an overview cross-sectional bright field transmission electron microscopy (BFTEM) image of the wear scar at the 1200th cycle after the final polishing of the FIB process. It can be found that the grains are refined below 1 μm with a lot of defects, such as dislocations. Figure 9b shows the magnified image from the rectangle in Figure 10a. It can be clearly found that the main crack firstly propagates along an 40° angle to the wear surface. Figure 10c shows the indexed SAED taken from the dashed circle in Figure 9b. The SAED shows an austenite structure with some substructures. Figure 10d shows the corresponding dark field transmission electron microscopy (DFTEM) image taken from the red site in Figure 10c. It can be clearly seen that the equiaxed grains and the lamellar grains exist near the wear surface.

**Figure 11:**
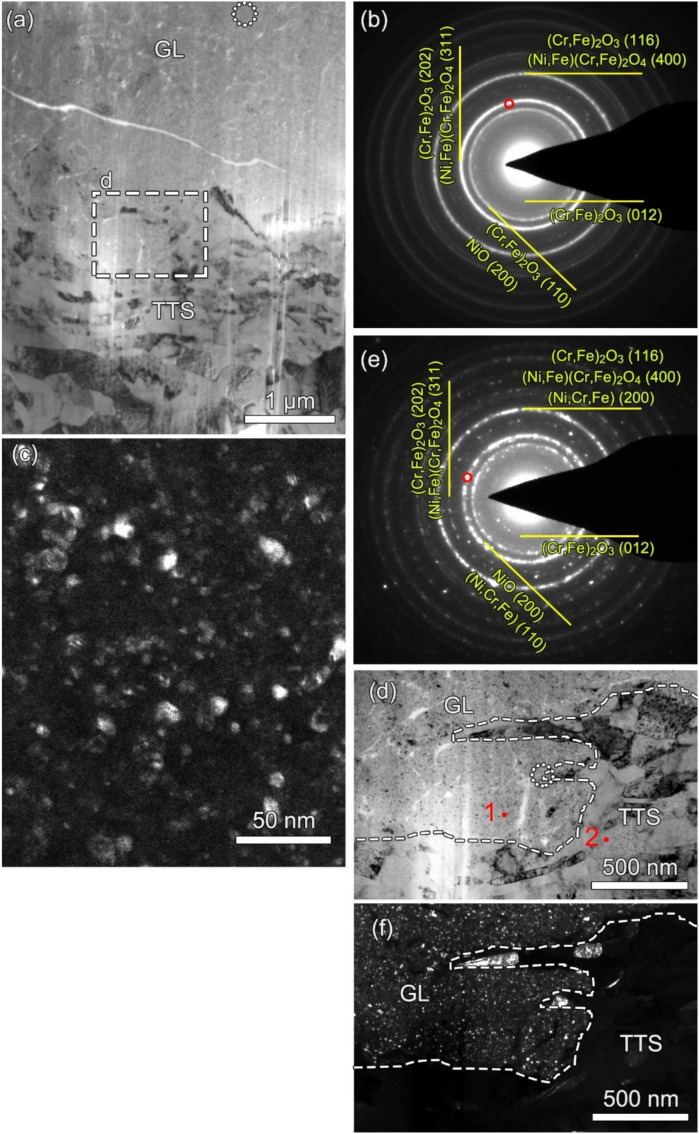
(**a**) An overview cross-sectional BFTEM image of the wear scar at the 6000th cycle after the final polishing of the FIB process. (**b**) The indexed SAED taken from the dashed circle in (**a**). (**c**) The corresponding DFTEM image taken from the red site in (**b**). (**d**) The magnified image from the rectangle in (**a**). (**e**) The indexed SAED taken from the dashed circle in (**d**). (**f**) The corresponding DFTEM image taken from the red site in (**e**).

Figure 11a shows an overview cross-sectional BFTEM image of the wear scar at the 6000th cycle after the final polishing of the FIB process. The gradient nanostructures can be observed in the subsurface, including the GL with a thickness of 2 μm and the TTS layer. The grain size gradually decreases as the microstructure changes from the TTS to GL. Although the GL seems to be compacted, there still are cracks and voids within the GL. Figure 11b shows the indexed SAED taken from the dashed circle in Figure 11a. The diffraction points are tightly connected, indicating that the grain size is ultrafine. Different oxides, such as the spinel type (FeCr_2_O_4_, NiCr_2_O_4_, NiFe_2_O_4_, Fe_3_O_4_), NiO, and the hematite type (Cr_2_O_3_ and Fe_2_O_3_), are found because the rings in the SAED for different oxides are superimposed, when compared to the X-ray diffraction (XRD) cards in the software. Figure 11c shows the corresponding DFTEM image taken from the red site in Figure 11b. It can be found that the average grain size in the GL is approximately 10 nm. Figure 11d shows the magnified image from the rectangle in Figure 11a. The TTS layer also contains the equiaxed grains and the lamellar grains. There are cracks and voids in the GL and at the interface between the GL and TTS layer. Figure 11e shows the indexed SAED taken from the dashed circle in Figure 11d. The oxides, just like in Figure 11b, and the austenite grains can be indexed. Figure 11f shows the corresponding DFTEM image taken from the red site in Figure 11e. The ultrafine-grained structure in the GL and the larger grains in the TTS layer can be clearly observed. Table 3 shows the EDS point analysis taken from Figure 11d for the chemical composition of the GL and TTS. It can be found that the GL is fully oxidized, however, as there is no oxygen in the TTS layer.

## 4. Discussion

Ft–D curves are very important, containing the dynamic characteristics obtained in a tangential fretting wear test. Based on the three-fretting regime definition method given by Zhou and Vincent [10,26,27], the fretting running status is the partial slip, when the Ft–D curves open to the elliptic, and the relative motion is coordinated by elasto-plastic deformation. All parallelogram Ft–D curves indicate the fretting running status of gross slip in the whole contact zone. The necessary condition of the mixed fretting regime (MFR) is that the fretting running status must be transformed between the partial slip regime (PSR) and gross slip regime (GSR). Based on the above analysis, with the gradual increase in the cycle number, the fretting running status undergoes a transition from partial slip and mixed stick-slip to final gross slip, as shown in Figure 2. 

Once the fretting starts, the original adsorption film and oxide film gradually rupture, resulting in direct metal–metal contact and the increase in actual contact area. Therefore, the coefficient of friction (COF) increases rapidly due to the surface adhesion and plastic deformation at the contact area [28,29]. The first drop in the COF is due to the changes in stress and strain with the occurrence of fatigue cracking, as shown in Figure 6a and Figure 7a. The continuous surface work hardening results in an increase in the brittleness of the surface, which caused the particles to peel off. A large number of particles accumulate to form a third body layer (TBL) on the surface, which act as a solid lubricant [30,31]. As the two-body to three-body transition occurs, the COF gradually decreases. Therefore, the second and third decreases in the COF are closely correlated with the evolution of the TBL.

As the fretting cycle goes on, the TBL is gradually transformed from a loose and uncompacted layer to form a stable oxide layer on the load-bearing areas with the effects of sufficient oxidation and mechanical action. A stable oxide layer on the top surface is called the glaze layer (GL) [32]. On the one hand, the formation of the GL is time- and temperature-dependent. For example, at high temperatures, even after a few minutes, a GL is formed on the surface of the alloys. At low temperatures, the GL takes longer periods to be established [33]. Furthermore, there is a threshold temperature of 220 °C above which a lubricating glaze layer is activated [34], and completely formed with its stabilized high lubrication properties, to lower the COF and wear volume. Also, the GL can be formed at ambient temperatures during the friction and wear due to the localized high flash temperature [35]. Therefore, the GL is first formed in a local site, at which the localized temperatures are high enough to induce thermal softening, creating a thin, physically homogeneous glaze. Then the GL is widely distributed on the whole contact surface. Based on the above analyses, it takes time to build up the GL. Therefore, the GL is formed at the 6000th fretting cycle, as shown in Figure 6b. The GL is also diffusely distributed in the company of loose wear debris. Once this local GL is established, the second drop-off in the COF occurs, together with a reduced wear rate, shown in Figure 2. As the GL is gradually formed on the whole contact surface (Figure 6c–e), the third drop in the COF takes place. The third descent process takes more time than the previous two. The COF decreases by 11% in the second process and 23% in the third process. This indicates that the GL, widely distributed throughout the contact surface, is more resistant to fretting wear. As shown in Figure 11, the GL actually consists of compacted ultrafine-grained oxide particles, which is consistent with a previous study [36]. The oxidation process is controlled by the diffusion of the oxygen, the substrate elements, and the thermodynamic nature of the relevant oxides [37,38]. All the elements in the alloy can be oxidized to form single component oxides, such as NiO, Cr_2_O_3_, and Fe_2_O_3_, or more complex oxides, such as NiCr_2_O_4_ [39,40], as shown in Figure 11b. 

With the increase in cycle number, the fretting runs from the PSR and MFR to the GSR. Correspondingly, the main damage mechanism of the first two regimes is fretting induced fatigue cracking (FIF), while the latter is fretting induced wear (FIW) [41]. The competition between FIF and FIW runs through the entire fretting wear process. At the 1200th cycle, the fatigue cracks are observed at the stick–slip region, shown in Figure 7a, Figure 8a and Figure 9a–c, which is due to high shear stress concentration at the stick–slip interface [42]. The wear mechanism is the combination of adhesive wear in the sticking region and abrasive and oxidation wear in the micro-slip zone. However, the FIF is absolutely dominant. With the cycle increase to 6000, a sudden increase in fatigue cracks indicates the competition between FIF and FIW. The wear mechanism shifts to be the combination of abrasive wear and oxidation, as shown in Figure 5 and Figure 6b. The fatigue cracking initiates at the contact center and edge and propagates along a sharp angle of about 15–45° to the base alloy, as shown in Figure 7b, Figure 8b and Figure 10b. It manifests that the velocity of crack propagation is higher than that of wear, and FIF wins the competition between FIF and FIW. As the cycle increases to 12,000, the surface pits are prominent with slightly weakened fatigue cracks, as shown in Figure 7c and Figure 8c. The competition between FIF and FIW seems to end in a draw. As the fretting cycle continues to increase, no cracking propagation to the base alloy can be found. At the same time, only some parallel cracks induced by delamination can be observed, as shown in Figure 7d and Figure 8d. This indicates that the wear velocity is obviously faster than that of FIF, and FIW achieves victory in the competition. 

A tribologically transformed structure (TTS) can be observed during these processes, as shown in Figure 10a and Figure 11a. It plays an important role in crack initiation and propagation and particle detachment by delamination [15,39,43]. The TTS is composed of nanograins, which can suppress fatigue crack initiation [44] but support delamination cracking [45]. The gain boundaries and dislocations are suitable pathways for crack propagation and oxygen permeation, as shown in Figure 8b–d, Figure 9c and Figure 10. When FIF and FIW compete with each other, the TTS also participates and plays an important role. It can arrest microcrack propagation, which causes microcracks to turn to the TTS layer [45] and changes the propagation direction. The dislocation interactions and the synergy of oxidation within the TTS layer support the formation of nano-cavities [46] which enhance crack initiation and propagation.

## 5. Conclusions

In this study, not only the subtle change of the coefficient of friction (COF) and wear volume were found, but microscopic damage was investigated to reveal the underlying wear mechanism with cycle number. Based on the experimental results and analyses, the following conclusions can be drawn:

1. The fretting behavior of alloy 690TT changed with the increase in cycle number. The fretting running status underwent a transition from partial slip and mixed stick-slip to final gross slip with the transformation of Ft–D curves from elliptic to the parallelogram. The relative motion was coordinated by elasto-plastic deformation in a partial slip regime (PSR) and plastic deformation in a gross slip regime (GSR).
2. Once the fretting started, the COF increased rapidly due to the surface adhesion and plastic deformation at the contact. The COF experienced three drops throughout the fretting process, which indicated the transformation from high-friction wear to low-friction wear. The first drop was due to the transition from two-body to three-body contact. The second drop was mainly due to the formation of localized glaze. The third drop was because the glaze was formed on the whole contact surface. The third drop took more time than the previous two.
3. The competition between fretting induced fatigue cracking (FIF) and fretting induced wear (FIW) ran throughout the entire fretting wear process. Before the 1.2 × 10^4^th cycle, the velocity of crack propagation was faster than that of wear, and FIF became the winner in the competition. As the fretting cycle continued to increase, the wear velocity was obviously faster than that of FIF, which indicated that FIW defeats FIF.
4. A tribologically transformed structure (TTS) played an important role in crack initiation and propagation and particle detachment by delamination. The TTS participated in the competition between FIF and FIW. The gain boundaries and dislocations in the TTS were suitable pathways for crack initiation and propagation and oxygen permeation.

## Figures and Tables

**Table 1:** Chemical composition of alloy 690TT and 304SS (wt.%).

Specimen	Element								
	Ni	Fe	Cr	C	Ti	Mn	Si	P	S
Alloy 690TT	Bal	11.6	29.9	0.025	0.30	0.25	0.33	0.086	0.0025
304SS	9.35	Bal	18.3	0.018	-	1.31	0.31	0.034	0.0025

**Table 2:** Mechanical properties of the alloys.

Specimen	Vickers Hardness (HV)	Yield Strength (MPa)	Tensile Strength (MPa)
Alloy 690TT	235	325	725
304SS	210	265	595

**Table 3:** Chemical composition of the GL and TTS taken from the EDS point analyses in Figure 10d (at%).

Location	Element			
	Ni	Fe	Cr	O
GL	43.58	7.40	18.58	30.44
TTS	66.31	9.70	23.98	-
